# Clinical Characteristics and Treatment Strategies for Group B Streptococcus (GBS) Infection in Pediatrics: A Systematic Review

**DOI:** 10.3390/medicina59071279

**Published:** 2023-07-09

**Authors:** Nawaf M. Alotaibi, Sharefa Alroqi, Abdulrahman Alharbi, Basil Almutiri, Manal Alshehry, Rinad Almutairi, Nada Alotaibi, Atheer Althoubiti, Ashwaq Alanezi, Nouf Alatawi, Hanan Almutairi, Munira Alhmadi, Rawan Almutairi, Mohammed Alshammari

**Affiliations:** 1 Department of Clinical Pharmacy, Northern Border University, Rafhaa 73213, Saudi Arabia; nawaf.al-otaibi@nbu.edu.sa; 2 Department of Clinical Pharmacy, Shaqra University, Al-Dawadimi 17472, Saudi Arabia; sharefaalroqi@gmail.com (S.A.); rinaad.1266@gmail.com (R.A.); nadasalotibi@gmail.com (N.A.); munira.alhmadi2@gmail.com (M.A.); rawansajdi@gmail.com (R.A.); 3 Department of Pharmaceutical Care, King Faisal Specialist Hospital & Research Centre, Al Madinah Al Munawwarah 42355, Saudi Arabia; aubdalrhman.f@gmail.com; 4 Alrazi Medical Company, Al-Qassim 56323, Saudi Arabia; basilbander@outlook.com; 5 Department of Clinical Pharmacy, King Khalid Hospital in IV & Nutrition TPN, Tabuk 32593, Saudi Arabia; manooola465@gmail.com; 6 Hokmaa Taif Medical Complex, Kingdom of Saudi Arabia, Taif 21944, Saudi Arabia; ath_414@hotmail.com; 7 College of Clinical Pharmacy, King Faisal University, Al-Ahasa 13890, Saudi Arabia; ashwaqalianezi@gmail.com; 8 Faculty of Pharmacy, University of Tabuk, Tabuk 71491, Saudi Arabia; noufmoh333@gmail.com (N.A.); hananxay@gmail.com (H.A.); 9 Department of Pharmaceutical Care, Northern Border Cluster, Arar 73311, Saudi Arabia

**Keywords:** antibiotics, infections, postpartum, prophylaxis, neonates

## Abstract

*Background and Objectives*: Group B streptococcus (GBS) is the leading cause of infections in neonates with high fatality rates. GBS is caused by the streptococcus bacterium known as streptococcus agalactiae, which is highly contagious and can be transmitted from pregnant women to infants. GBS infection can occur as an early onset or late-onset infection and has different treatment strategies. Antibiotics are effective in treating GBS infections at early stages. The aim of this systematic review was to summarize the clinical characteristics and treatment strategies for GBS, with a focus on antibiotics. *Material and Methods*: The findings of this review were reported in accordance with the PRISMA 2020 guidelines and a flow diagram of the study selection process, a summary of the included studies, a description of the study characteristics, a summary of the results, a discussion of the implications of the findings, and a conclusion are included. Overall, the authors followed a rigorous methodology to ensure that this review is comprehensive and inclusive of relevant studies on GBS infection and its treatment. *Results*: Overall, 940 studies were reviewed and only the most relevant 22 studies were included in the systematic review. This review describes the characteristics of patients in different studies related to early onset GBS disease and presents various treatment strategies and outcomes for GBS infection in pediatrics. The studies suggest that preventive measures, risk-based intrapartum antibiotic prophylaxis, and maternal vaccination can significantly reduce the burden of GBS disease, but late-onset GBS disease remains a concern, and more strategies are required to decrease its rate. Improvement is needed in the management of the risk factors of GBS. A conjugate vaccine with a serotype (Ia, Ib, II, III, and V) has been proven effective in the prevention of GBS in neonates. Moreover, penicillin is an important core antibiotic for treating early onset GBS (EOD). *Conclusions*: This systematic review summarizes the treatment comparison for GBS infections in neonates, with a primary focus on antibiotics. IAP (intrapartum antibiotic prophylaxis) according to guidelines, antenatal screening, and the development of a conjugate vaccine may be effective and could lower the incidence of the disease.

## 1. Introduction

Group B streptococcus (GBS), a Gram-positive bacterium also known as streptococcus agalactiae, is a type of bacterial infection, which includes sepsis, pneumonia, and meningitis, that affects newborns and infants worldwide [1,2,3]. GBS is a significant cause of neonatal morbidity and mortality, and early identification and treatment are essential for improving outcomes [1,2,3,4,5,6]. The frequency was estimated to be 0.49 cases per thousand live births, according to a recent systematic study [7]. According to estimates, this occurrence causes more than 90,000 infant fatalities every year with the death count ranging from 35,000 to 170,000 [8]. Moreover, the mean fatality ratio for neonates with invasive GBS illness was 9.6% in one systematic review and meta-analysis, and it was approximately threefold greater in low-income countries, at 12.6%, than in high-income countries, at 4.6% [9]. Additionally, 32% of newborns with GBS meningitis who survive the disease display neurodevelopmental damage 18 months after the infection, with 18% having moderate-to-severe neurodevelopmental damage [10]. The long-term neurological impairment caused by GBS meningitis is significant. It is also identified in one out of every five women’s guts and genital tracts, with one-third of these mothers transmitting the bacteria to their infants during pregnancy or birth [8]. Early onset illness may develop after transmission from infected mothers to their infants before or during birth [11,12]. Stillbirths, preterm births, and puerperal sepsis are all significantly influenced by GBS [8,13]. Vertical, nosocomial, or community transmission are all possible causes of late-onset illness [3,7,14].

Several treatment strategies have been proposed for GBS infection in pediatrics, including prophylactic antibiotics, intravenous immunoglobulin, and supportive care. Intrapartum prophylactic antibiotics are the most commonly used treatment strategies for GBS infection in pediatrics [15]. Antibiotics can be administered to pregnant women four hours before delivery to prevent the vertical transmission of early onset GBS illness to the newborn [16]. In infants who have already developed GBS infection, antibiotics can also be used to treat the infection [17]. Such tactics are either based on swab-based screening to identify at-risk women or on the existence of clinical risk indicators or symptoms [18,19,20]. Nonetheless, intrapartum antibiotic prophylaxis does not shield against conditions related to late onset illness. Antibiotics used as a preventative measure during childbirth have significantly decreased the incidence of early onset infant GBS [21]. However, concerns have been raised regarding the overuse of antibiotics, which can lead to the development of antibiotic-resistant strains of bacteria [22]. Resistance to erythromycin and clindamycin has developed significantly over the last 20 years but may differ by region [23,24]. Additionally, the use of antibiotics in newborns can disrupt the developing microbiome and increase the risk of other infections [25,26,27]. In order to develop the best preventative and therapeutic measures, it is important to understand the clinical conditions and local antimicrobial medication resistances of GBS strains [28].

Intravenous immunoglobulin (IVIG) is an alternative treatment strategy that has been proposed for GBS infection in pediatrics [29]. IVIG is a pooled human immunoglobulin preparation that contains antibodies against a variety of pathogens, including GBS. IVIG has been shown to be effective in reducing the risk of GBS infection in neonates and improving outcomes in infants with GBS infection [30]. However, IVIG is expensive and not widely available, and there are limited data on its long-term safety. Moreover, supportive care may include the use of oxygen therapy, intravenous fluids, and the monitoring of vital signs. Supportive care is important to stabilize the infant’s condition and manage any complications that may arise. However, supportive care alone may not be sufficient to treat the underlying infection, and the infant may require additional interventions [31].

There is a need for a systematic review of the available literature to compare the clinical characteristics and various treatment strategies for GBS infections in pediatrics. Such a review can help healthcare providers make informed decisions regarding the most effective and safe treatment strategy for their patients. The review will consider studies that evaluate the efficacy and safety of prophylactic antibiotics, IVIG, and supportive care in the treatment of GBS infection in pediatrics. This review will also assess the impact of these treatments on long-term outcomes, such as neurodevelopmental outcomes and the development of antibiotic resistance.

## 2. Materials and Methods

The present systematic review was conducted in accordance with the Preferred Reporting Items for Systematic Reviews and Meta-Analyses (PRISMA) 2020 guidelines.

Information sources:

A comprehensive search strategy was developed, and the databases of PubMed, Google scholar, Cochrane Library, and the Web of Science were searched. The search strategy was developed using medical subject headings (MeSH) and keywords related to Group B streptococcus, pediatrics, antibiotics for GBS, and treatment strategies, e.g., “Group B Streptococcus” OR “GBS” OR “Streptococcus agalactiae” AND “neonates” OR “newborns” OR “infants” AND “treatment” OR “therapy” OR “management” OR “antibiotics” OR “prophylaxis”. The databases were searched from their inception up to the present, specifically from the year 2000 to 2022.

Study selection:

The searched studies were scrutinized by two reviewers independently by reading the study title and abstract of that study. Studies were considered eligible if they met the following criteria: (1) included participants under the age of 18 with GBS infection; (2) evaluated one or more treatment strategies for GBS infection; (3) reported clinical outcomes; and (4) were published in English. The full text studies for GBS infections and their treatment were included in this systematic review. The retrieved articles were merged, and duplicates were excluded. In addition, the references of the studies were reviewed extensively to search for other potential studies. The studies published with different study designs were included in this review. The eligibility criteria for the study selection in this systematic review included both randomized controlled trials (RCTs) and observational studies. This approach allowed for a comprehensive analysis of the available evidence on the clinical characteristics and treatment strategies for Group B streptococcus (GBS) infection in neonates. By considering both RCTs and observational studies, we aimed to capture a wide range of data and perspectives to provide a comprehensive overview of the topic. The ethical approval codes were considered as a criterion for inclusion in the manuscript.

Data extraction:

The data included in this review were retrieved from the text of articles, tables, figures, and results section. Two reviewers independently extracted data from the included studies using a standardized data extraction form. The following data were extracted: study characteristics (e.g., author, year of publication, study design, sample size), participant diagnostic factors (e.g., hematological parameters, biomarkers, blood cultures), mother-related risk factors (e.g., PROM, gestational age at delivery, mode of delivery, delivery <37 weeks of gestation, low birth weight), neonate-related risk factors (e.g., low Apgar score, resuscitation at birth, need for artificial ventilation), treatment (e.g., drug, dose, duration), clinical outcomes, therapy endpoints (e.g., length of NICU stay, recovery/success rate, change of antibiotics, death), and treatment recommendations. Any discrepancies were resolved through discussion or with the involvement of a third reviewer.

Reporting:

The findings of this review were reported in accordance with the PRISMA 2020 guidelines and a flow diagram of the study selection process, a summary of the included studies, a description of the study characteristics, a summary of the results, a discussion of the implications of the findings, and a conclusion are included. Overall, the authors followed a rigorous methodology to ensure that their review was comprehensive and inclusive of relevant studies on GBS infection and its treatment.

## 3. Results

**Figure 1:**
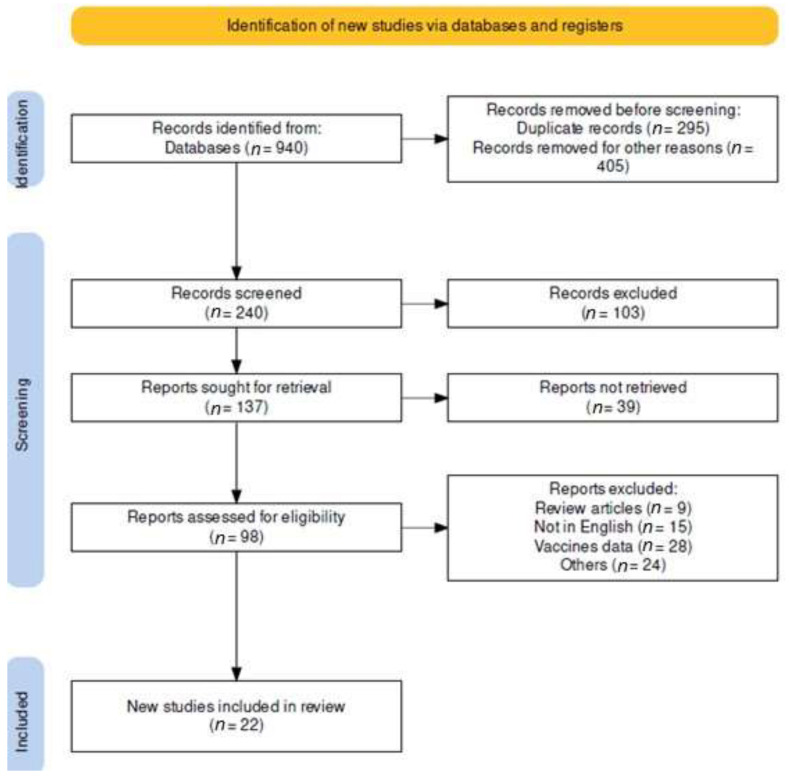
PRISMA flow diagram of included studies.

A total of 940 relevant published studies were retrieved from online search databases (Figure 1). Of these, 295 studies were removed because they were duplicates and 405 because of other reasons. The inclusion criteria for this systematic review were as follows: the studies had to be published between 2000 and 2022, and they had to provide information about the clinical characteristics and use of antibiotics for the prophylaxis and treatment of the group B streptococcal (GBS) disease. After further screening, 142 studies were removed. Of the remaining 98 studies, 9 review articles, 15 non-English papers, and 28 vaccine-related studies were excluded, as well as a further 24 for other reasons, i.e., because they had inadequate data or because they evaluated interventions other than the inclusion criteria. The final set of 22 studies included in this systematic review met all the inclusion criteria.

Table 1 provides the characteristics of patients involved in different studies related to early onset group B streptococcal (GBS) disease. The studies in the table include retrospective and prospective cohort studies, randomized controlled trials and surveillance studies. Studies had smaller sample sizes, ranging from 50 in a prospective case–control study to more than a million in a cohort study. Diagnostic factors for GBS infection, such as blood cultures, biomarkers, and hematological parameters, are also mentioned in the table. Other factors that may increase the risk of GBS infection in newborns, such as the premature rupture of membranes (PROM), low birth weight, and the need for artificial ventilation, are also noted. Some studies examined both mother and neonate-related risk factors, while others focused only on neonatal factors. The findings related to maternal-related risk factors and diagnostic factors were less consistent across studies. Several studies found that preterm delivery and low birth weight were significant risk factors for GBS infection in newborns. However, some studies found conflicting results on the association between GBS colonization in mothers and the risk of infection in newborns. Moreover, the studies had different diagnostic factors. Some of the studies used only blood cultures to diagnose GBS infection, while others used a combination of blood cultures, vaginal cultures, and rectal cultures.

Table 2 presents various treatment strategies for group B strep (GBS) infection in pediatrics along with the therapy outcomes. The table includes information on the length of NICU stay, recovery/success rate, change of antibiotics, death, treatment, clinical outcomes, and treatment recommendations for each strategy. One of the studies in the table reported that the prevalence of meningitis was higher in late-onset disease (LOD) than in early onset disease (EOD). Another study found that the use of point-of-care rapid tests in labor and risk factor-based IAP reduced GBS cases. Additionally, the use of a hexavalent vaccine, including serotypes Ia, Ib, and II-V, was recommended to cover all types of strains. Another study in the table reported that the incidence of EOD decreased with IAP but the incidence of LOD remained the same. Vaccines to prevent LOD were recommended. In addition, the incidence of EO-GBS decreased with universal screening and IAP. The table also shows that the use of IAP with penicillin, ampicillin, cefazolin, clindamycin, and/or vancomycin for ≥4 h reduces the risk of neonatal sepsis. However, it was found to increase BMI. Another study found that the use of IAP helps in PPROM and PROM by reducing EOGBS. Overall, the studies in the table suggest that preventive measures, risk-based IAP, and maternal vaccination can significantly reduce the burden of GBS disease. However, LOD remains a concern, and more strategies are required to decrease its rate. Finally, the table suggests that a GBS conjugate vaccine with serotypes Ia, Ib, II, III, and V can help prevent infection. In a case–control study conducted over a 37-month period, 242 cases of invasive GBS infection were recorded from participating institutions. Of these cases, 138 (57%) had their caretakers’ consent for their participation. Additionally, 305 (25%) of the parents of the 1220 matched controls consented. Cases and controls were closely matched in terms of birth weight (cases: range 500–4840 g; median 3246 g; controls: 677–4680 g; median 3200 g). Additionally, six sets of twins were reported among the controls and two combinations of affected twins among the cases. During the monitoring period, a second episode of GBS occurred in three individuals (2.2%). The most frequently prescribed antibiotic combinations for the clinical treatment of GBS-infected infants were gentamicin and penicillin (39%), cefotaxime (13%) alone, and penicillin and cefotaxime (7%). Among the 138 infants, 109 (79%) underwent lumbar punctures. A total of 21 different antibiotic combinations were used. Antibiotic treatment for sepsis lasted an average of 9 days (median 8, range 4–21), while it took an average of 15.7 days for meningitis (median 14, range 7–35).

**Figure 2:**
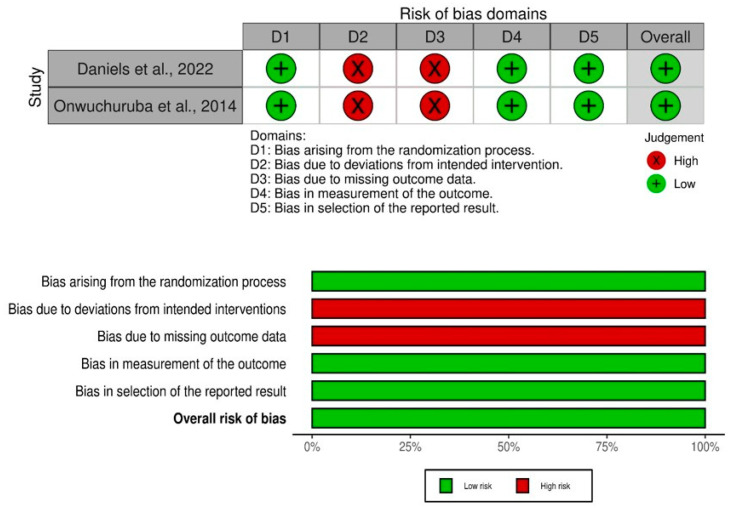
Quality assessment of randomized controlled trials [33,43].

The quality assessment of the included studies was conducted using two different tools. The Newcastle–Ottawa Scale (NOS) was used for cohort studies, which assesses data based on three subscales: selection, comparability, and outcomes. For randomized controlled trials (RCTs), the Cochrane bias tool was employed. This tool evaluates the risk of bias in each study across various domains, such as random sequence generation, allocation concealment, blinding, incomplete outcome data, and other sources of bias. The judgments for each domain were categorized as “high risk”, “low risk”, or “unclear.” Two authors independently assessed each article, and any discrepancies were resolved through discussion and consensus. The detailed results of quality assessment are given in Table 3, Table 4 and Table 5 and Figure 2.

## 4. Discussion

In this systematic review, we focused on the clinical characteristics and therapeutic approaches for GBS infections in newborns. GBS is the most prevalent source of severe illness in neonates, young children, and pregnant women with impaired immune systems [32]. The majority of the case–control and observational studies on the assessment of risk factors for early-onset illness in neonates were carried out in the United States [51]. The most common risk factors are preterm birth, low birth weight, protracted membrane rupture, intrapartum fever, young mother age, Black ethnic group, prior delivery of an affected infant, and low levels of anti-capsular antibody [52]. Although the risk factors for maternal, nosocomial, and breast milk sources of late-onset and early-onset GBS have been documented, there is currently little knowledge of these risk factors [11]. GBS infection can cause a variety of serious complications, including sepsis, meningitis, and pneumonia. The mortality rate from GBS infection is highest among preterm infants [8,36,50,53]. Studies suggest that there are a number of things that can be done to reduce the risk of GBS infection in infants. These include screening pregnant women for GBS colonization, treating pregnant women with GBS colonization with antibiotics, and delivering infants early if the mother has GBS colonization [6,7,20].

The increased use of IAP may have an impact on the incidence of early-onset non-GBS sepsis and the emergence of antibiotic resistance in Gram-negative bacteria that colonize the infant at birth. Some evidence suggests that this may be the case; however, a lot of research has been conducted on very small cohorts or institutions. Preterm infants are more likely to have gram-negative sepsis, which most frequently involves Escherichia coli, and this may have an impact on their premature birth. Increasing numbers of premature infants are developing E. coli sepsis according to some researchers. A significant multicenter study that looked at 141,000 newborns over the course of four years found no rise in the prevalence of non-GBS early onset neonatal infection [19]. In Australia, the routine utilization of prophylactic intrapartum antibiotics and antenatal testing for GBS carriage was advised 20 years ago; however, the widespread use of intrapartum antibiotics did not begin until the 1990s, following the publishing of comparable recommendations in the United States. By 1999, 11 out of 11 Australian obstetric institutions had obstetric policies for the prevention of early onset GBS infection, up from 3 out of 9 in 1992–1993. The decision to administer intrapartum chemoprophylaxis was made in 62 of 64 (97%) of the obstetric hospitals surveyed in Victoria, Australia (48 rural and 16 metropolitan), utilizing either a screening (48 hospitals) or a risk-based (14 hospitals) strategy between 1997 and 1998 [54].

Penicillin is the suggested antibiotic for GBS prophylaxis due to its limited spectrum of antibacterial action, even if ampicillin is a suitable substitute. There are no known cases of GBS resistance to penicillin or ampicillin [49]. Penicillin allergies are reported by 10% of the population. For GBS prevention, historically, individuals who reported a penicillin allergy were given clindamycin or erythromycin [41]. Several high-income nations, like the UK, are still unsure of the relative merits and drawbacks of universal screening and instead opt for a risk-based strategy in which all pregnant women with risk factors are given intrapartum antibiotic prophylaxis to prevent GBS infection in their infants before it has even begun to manifest. When sepsis is suspected or diagnosed, newborns are closely watched for indications of infection and given medicines if necessary [6]. The incidence of GBS resistance to erythromycin and clindamycin is rising in the United States, with rates estimated to be 3–15% for erythromycin and 7–25% for clindamycin. Clinical investigations showed that intrapartum intravenous ampicillin or penicillin was very effective at preventing invasive early-onset GBS disease in mothers at risk of transferring the illness to their newborn in the 1980s [54]. A significant reduction in incidence (0.5/1000 vs. 1.0/1000 live births) was seen in the penicillin group compared to a control cohort that only received intrapartum antibiotics for maternal infection [50,53].

There were a few limitations with this systematic review. Firstly, no quantitative analyses were performed, as the objective of this systematic review was just to highlight the clinical characteristics and treatment strategies for GBS infections. Moreover, the heterogeneity of the included studies in terms of study design, sample size, and outcome measures may limit the ability to conduct a meta-analysis or draw definitive conclusions. Secondly, there were limited number of studies included due to the minimal availability of the literature to treat GBS with antibiotics. The search was limited to specific databases, and the exclusion of other databases, such as Scopus, may have resulted in the omission of relevant studies. Moreover, the Scimago Q1 database was not the inclusion criteria for all studies in the specific medical field, and this may limit the comprehensiveness of the included literature in our study. Lastly, the quality assessment tools used for evaluating the included studies, such as the Newcastle–Ottawa Scale (NOS) and Cochrane bias tool, have their own limitations and subjectivity, which may affect the overall assessment of study quality. It is important to consider these limitations when interpreting the results of the study and to recognize the need for further research to address these limitations and provide more comprehensive insights into the management of GBS infections in neonates.

## 5. Conclusions

This systematic review summarizes the treatment comparison for GBS infections in neonates with a primary focus on antibiotics. Postpartum antibiotics prophylaxis in pregnant women and neonates may be effective and lower the incidence of the disease. Moreover, the vaccinations to treat GBS are much more effective. Furthermore, there is a pressing need to identify more treatment strategies for GBS infections in infants.

## Figures and Tables

**Table 1:** Characteristics of patients.

Author and Year	Study Design	Sample Size	Diagnostic Factors			Risk Factors							
						Mother-Related				Neonate-Related			
			Hematological Parameters	Biomarkers	Blood Cultures	PROM	Gestational Age at Delivery	Mode of Delivery	Delivery <37 Weeks of Gestation	Low Apgar Score	Resuscitation at Birth	Need for Artificial Ventilation	Low Birth Weight
Andersen et al., 2022 [32]	Prospective cohort study	212 infants; 129 EOD 83 LOD	NA	NA	Yes	Yes	Yes	NA	Yes	Yes	Yes	Yes	Yes
Daniels et al., 2022 [33]	Parallel group cluster randomized trial	Rapid test units; 722 mothers (749 babies), Usual care units; 906 mothers (951 babies)	NA	NA	Yes	Yes	Yes	Yes	Yes	NA	NA	NA	NA
Hong, Z., et al., 2022 [34]	Retrospective Cohort study	2909 mother–child pair	NA	NA	NA	Yes	Yes	Yes	No	NA	NA	NA	No
Koebnick et al., 2021 [35]	Retrospective cohort study	223,431 infants of 177,666 mothers	NA	NA	Yes	NA	Yes	Yes	Yes	NA	NA	NA	No
Mynarek, M., et al., 2021 [36]	Cohort study	625 infants	NA	NA	Yes	Yes	Yes	Yes	Yes	Yes	NA	Yes	Yes
Baeringsdottir et al., 2021 [37]	Surveillance study	105 infants	NA	NA	Yes	Yes	Yes	Yes	Yes	Yes	NA	Yes	Yes
Cho et al., 2019 [20]	Retrospective analysis	9535 pregnant women and their 9845 babies	Yes	Yes	Yes	Yes	Yes	Yes	Yes	Yes	Yes	Yes	Yes
Yeo, K.T., et al., 2019 [38]	Cohort study	1,023,392 infants, 1206 with GBS	NA	Yes	NA	Yes	Yes	Yes	Yes	Yes	NA	Yes	Yes
O’Sullivan et al., 2019 [39]	Prospective surveillance study	856 infants	NA	NA	NA	Yes	Yes	NA	Yes	NA	NA	NA	Yes
Santhanam et al., 2017 [11]	Retrospective case–control study	54 cases, 216 controls	NA	NA	NA	Yes	Yes	Yes	NA	Yes	Yes	NA	Yes
Toyofuku et al., 2017 [40]	Prospective longitudinal cohort study	730 mothers and infants	NA	NA	NA	NA	NA	NA	NA	NA	NA	NA	NA
Briody et al., 2016 [41]	Retrospective cohort study	165 women	Yes	Yes	Yes	Yes	Yes	Yes	No	Yes	Yes	Yes	Yes
Onwuchuruba et al., 2014 [42]	Randomized controlled trials	55 patients	NA	NA	NA	NA	Yes	NA	NA	Yes	NA	NA	Yes
Turrentine et al., 2013 [43]	Retrospective cohort study	4782 women	Yes	NA	Yes	Yes	Yes	NA	NA	NA	NA	Yes	Yes
Stoll et al., 2011 [44]	Prospective surveillance (2006–2009)	~400,000 live births at NICHD, NRN	NA	NA	Yes	Yes	Yes	Yes	Yes	NA	Yes	Yes	Yes
Daniels, J., et al., 2011 [19]	Diagnostic test accuracy study	1400 women	NA	NA	NA	Yes	NA	Yes	Yes	NA	NA	NA	NA
Van Dyke et al., 2009 [45]	Retrospective cohort study	7691 live births 254 infants with GBS	NA	NA	NA	Yes	Yes	Yes	Yes	NA	NA	NA	NA
Heath et al., 2009 [46]	Retrospective case–control study	138 cases, 305 controls	NA	NA	Yes	Yes	Yes	Yes	Yes	Yes	Yes	Yes	Yes
Jauréguy et al., 2004 [47]	Prospective case–control study	50 infants	NA	NA	NA	AN	Yes	Yes	Yes	NA	NA	NA	Yes
Schrag et al., 2002 [48]	Multistate retrospective cohort study	312 infants with EOD-GBS	NA	NA	NA	Yes	Yes	NA	Yes	NA	NA	NA	NA
Oddie and Embleton, 2002 [49]	Prospective case–control study	37 cases, 147 controls	NA	NA	Yes	Yes	Yes	Yes	Yes	NA	Yes	NA	NA
Schrag et al., 2000 [50]	Active, population-based surveillance 1993–1998	7867 infants	NA	NA	Yes	NA	Yes	NA	Yes	NA	NA	NA	NA

**Table 2:** Treatment strategies for group B strep (GBS) infection in pediatrics.

Author and Year	Therapy Outcomes				Treatment	Clinical Outcomes	Treatment Recommendations
	Length of NICU Stay	Recovery/Success Rate	Change of Antibiotics	Death			
Andersen et al., 2022 [32]	NA	NA	NA	Yes	Antibiotic for 7–16 days. Respiratory support. Circulatory support.	Prevalence of meningitis was higher in LOD than in EOD. Strain III/CC17 responsible for most GBS infections.	Multimodal surveillance of infant GBS strains. Hexavalent vaccine including serotype Ia, Ib, and II-V to cover all types of strains
Daniels et al., 2022 [33]	NA	Yes	NA	Yes	IAP according to national recommendations, benzyl penicillin as first choice	Neonates born to women in the rapid test units had a significantly lower risk of receiving antibiotics.	Use of point-of-care rapid test in labour and risk factor-based IAP to reduce GBS.
Hong, Z. et al., 2022 [34]	NA	Yes	Yes	NA	IAP according to national guidelines.	Vaginal delivery and GBS-IAP increase the risk of AD in children.	NA
Koebnick et al., 2021 [35]	NA	NA	Yes	No	IAP with penicillin G, ampicillin, cefazolin, clindamycin, and/or vancomycin for ≥4 h. SSIP with cefazolin	IAP decreases the risk of neonatal sepsis but increases BMI.	Alternative of IAP.
Mynarek, M., et al., 2021 [36]	Yes	Yes	NA	Yes	IAP	EOD incidence decreased by IAP.	Antenatal screening for all women and vaccination to reduce LOD and VLOD.
Baeringsdottir et al., 2021 [37]	Yes	NA	Yes	Yes	IAP with penicillin, ampicillin–clavulanic acid, ampicillin, cefazolin and erythromycin with gentamicin. EAT with ampicillin-gentamicin or cefotaxime to infants. Ampicillin, gentamicin, cefotaxime, netilmicin, penicillin to treat GBS	Incidence of EOD decreased, but of LOD remained same	Vaccines to prevent LOD.
Cho et al., 2019 [20]	Yes	NA	NA	No	IAP ≥ 4 h with penicillin, ampicillin, or cefazolin	Universal screening and IAP decreased neonatal EOD GBS	Strategies required to decrease the rate of LOD GBS.
Yeo, K.T., et al., 2019 [38]	Yes	Yes	NA	Yes	IAP	Overall reduction in EOGBS cases.	NA
O’Sullivan et al., 2019 [39]	NA	NA	NA	Yes	NICE guidelines antibiotics for EO infection, IAP according to CDC guidelines	EOD overall burden decreased, LOD burden remained the same.	Pentavalent conjugate vaccine (containing serotypes Ia, Ib, II, III, V) to prevent LOD.
Santhanam et al., 2017 [11]	NA	NA	No	Yes	Risk factor-based IAP therapy	IAP helps in PPROM and PROM in reducing EOGBS.	Avoid multiple vaginal examinations. Risk based IAP and newborn evaluation for EOS in middle-low-income countries.
Toyofuku et al., 2017 [40]	NA	NA	NA	NA	IAP	EOD-GBS reduced but LOD-GBS remained same	Trivalent GBS vaccine (CRM197-conjugated capsular polysaccharides of GBS serotype Ia, Ib, and III)
Briody et al., 2016 [41]	Yes	Yes	Yes	Yes	IAP with penicillin or cefazolin. Inappropriate use of erythromycin clindamycin vancomycin in penicillin allergy.	No specific differences between two groups.	IAP according to national guidelines.
Onwuchuruba et al., 2014 [42]	NA	Yes	NA	NA	Vancomycin dose; Phase I trial; 1 g every 12 h Phase II trial; 15 mg/kg every 12 h Phase III trial; 20 g/kg every 8 h	Vancomycin levels above the accepted MIC break point of 1 mg/mL for group B streptococcus were achieved in all.	20 mg/kg IV every 8 h (MID 2 g) to achieve newborn therapeutic level.
Turrentine et al., 2013 [43]	Yes	NA	NA	NA	IAP with penicillin. Ampicillin and gentamicin for <48 h or until blood cultures are negative in infants.	Significant reduction in GBS colonized newborns with IAP ≥ 4 h.	IAP for ≥4 h.
Stoll et al., 2011 [44]	Yes	Yes	Yes	Yes	IAP with ampicillin, gentamicin erythromycin. EAT with ampicillin, gentamicin, cefotaxime, vancomycin to GBS infants	Reduction in mortality by 16%	Strategies to reduce preterm birth
Daniels, J., et al., 2011 [19]	NA	NA	NA	NA	IAP	Neonatal GBS colonization rates decreased by PCR screening and IAP ≥ 4 h.	Intrapartum PCR testing and IAP ≥ 4 h.
Van Dyke et al., 2009 [45]	NA	NA	NA	NA	IAP with penicillin and ampicillin. Clindamycin in penicillin allergy patients.	Decline in EOD GBS after the implementation of universal antenatal screening.	Development of vaccines against GBS
Heath et al., 2009 [46]	Yes	Yes	Yes	Yes	Risk factor-based IAP. Penicillin-gentamicin being the most common, followed by cefotaxime alone and cefotaxime-penicillin.	Maternal, birth and neonatal factors are significantly associated with EOGBS disease and longer hospital stays.	IAP and GBS vaccination. Appreciable use of hospital resources for the management of GBS disease.
Jauréguy et al., 2004 [47]	NA	NA	NA	NA	2 g amoxicillin IV at labor and 1 g IV every 4 h till delivery.	Intestinal bacterial colonization was slightly delayed due to IAP.	Further evaluation required.
Schrag et al., 2002 [48]	NA	NA	NA	NA	IAP	Prenatal screening for GBS and IAP lowers the risk of EOD	Routine screening of GBS during pregnancy and IAP.
Oddie and Embleton, 2002 [49]	NA	NA	NA	Yes	IAP	Reduction in EOD GBS by using IAP.	Application of PHLS’s recommendations. PROM as an important risk factor for early stage diagnosis.
Schrag et al., 2000 [50]	NA	NA	NA	Yes	IAP	Reduction in GBS cases	GBS vaccine

**Table 3:** Quality assessment of cohort studies.

	Selection				Comparability	Outcomes		
References	Representative of Exposed Studies ^a^	Selection of Non-Exposed ^b^	Ascertainment of Exposure ^c^	Demonstration of Outcome ^d^	Comparability of Cohort Studies on Basis of Design ^e^	Assessment of Outcomes ^f^	Adequacy of Follow-Up ^g^	Quality Score
Andersen et al., 2022 [32]	*	*	*	*	*	*	*	7
Hong, Z. et al., 2022 [34]	*	*	*	*	*	**	*	8
Koebnick et al., 2021 [35]	*	*	*	*	*	**	*	8
Mynarek, M., et al., 2021 [36]	*	*	*	*	*	**	*	8
Baeringsdottir et al., 2021 [37]	*	*	*	*	*	*	*	7
Cho et al., 2019 [20]	*	*	*	*	*	**	*	8
Yeo, K.T., et al., 2019 [38]	*	*	*	*	**	**	*	9
Toyofuku et al., 2017 [40]	*	*	*	*	*	**	*	8
Briody et al., 2016 [41]	-	*	*	*	*	*	*	6
Turrentine et al., 2013 [42]	*	*	*	*	-	*	*	6
Van Dyke et al., 2009 [43]	*	*	*	*	**	*	*	8
Schrag et al., 2002 [48]	*	*	*	*	**	**	*	9

a: * = truly representative or somewhat representative of average in target population. b: * = drawn from the same community. c: * = secured record or structured review. d: * = Yes, - = No. e: * = study controls for age, gender, and other factors. f: * = record linkage or blind assessment. ** = both. g: * = follow-up of all subjects.

**Table 4:** Quality assessment of case–control studies.

	Selection				Comparability		Exposure			
References	Case Definition Adequate	Representativeness of the Cases	Selection of Controls	Definition of Controls	Main Factors	Additional Factors	Ascertainment of Exposure	Same Method of Ascertainment of Case and Controls	Non-Response Rate	Final Score
Santhanam et al., 2017 [11]	Yes	Yes	Yes	Yes	Yes	Yes	Yes	Yes	No	8
Heath et al., 2009 [46]	Yes	Yes	Yes	Yes	Yes	Yes	Yes	Yes	Yes	9
Jauréguy et al., 2004 [47]	Yes	Yes	Yes	Yes	Yes	No	No	No	No	5
Oddie and Embleton, 2002 [49]	Yes	Yes	Yes	No	Yes	No	Yes	Yes	No	6

**Table 5:** Risk of bias assessment for randomized controlled trials.

Study	Random Sequence Generation	Allocation Concealment	Blinding of Participants and Personnel	Blinding of Outcome Assessment	Incomplete Outcome Data	Selective Reporting	Other Bias
Daniels et al., 2022 [33]	Low risk	High risk	High risk	High risk	High risk	Low risk	Low risk
Onwuchuruba et al., 2014 [42]	Low risk	High risk	High risk	High risk	Low risk	Low risk	Low risk

## Data Availability

Not applicable.
